# Post-tensioning of glass beams: Analytical determination of the allowable pre-load

**DOI:** 10.1007/s40940-021-00150-0

**Published:** 2021-03-30

**Authors:** Jagoda Cupać, Christian Louter, Alain Nussbaumer

**Affiliations:** 1 Institute of Building Construction, Technische Universität Dresden, August-Bebel-Straße 30, 01219 Dresden, Germany; 2 Resilient Steel Structures Laboratory (RESSLab), School of Architecture, Civil and Environmental Engineering (ENAC), École Polytechnique Fédérale de Lausanne (EPFL), GC B3 495, Station 18, 1015 Lausanne, Switzerland

**Keywords:** Post-tensioned glass beam, Pre-load introduction, Failure modes, Analytical model, Numerical model, Parametric study

## Abstract

The effectiveness of post-tensioning in enhancing the fracture resistance of glass beams depends on the level of compressive pre-stress introduced at the glass edge surface that will in service be exposed to tensile stresses induced by bending. Maximum pre-load that can be applied in a post-tensioned glass beam system, yielding maximum compressive pre-stress, is limited by various failure mechanisms which might occur during post-tensioning. In this paper, failure mechanisms are identified for a post-tensioned glass beam system with a flat stainless steel tendon adhesively bonded at the bottom glass edge, including the rupture of the tendon, glass failure in tension and adhesive/glass failure in the load introduction zone. Special attention is given to the load introduction failure given that the transparent nature of glass limits the use of vertical confinement usually applied in concrete. An analytical model for determination of the allowable pre-load in post-tensioned glass beams is proposed, based on the model applied for externally post-tensioned concrete beams. The model is verified with the results of a numerical model, showing good correlation, and applied in a parametric study to determine the influence of various beam parameters on the effectiveness of post-tensioning glass beams.

## Introduction

Post-tensioned glass beams are hybrid structural components in which a ductile tendon is applied on a standard glass section to enhance its in-plane bending behaviour. The tendon introduces compressive pre-stress into the glass and thus compensates for the rather low resistance of glass in tension. A number of studies have investigated various methodologies of post-tensioning applied to glass beams, demonstrating significantly enhanced structural performance in bending in terms of initial fracture resistance and redundancy in the post-fracture state (Bos et al. 2004; Schober et al. 2004; Débonnaire 2013; Louter et al. 2013; Jordão et al. 2014; Louter et al. 2014; Engelmann and Weller 2019; Cupać et al. 2021). These studies have generally focused on the structural behaviour of post-tensioned beams in bending, which have been investigated experimentally and through numerical modelling, where particular attention has been given to the modelling of the brittle fracture of glass (Bedon and Louter 2016, 2017).

**Fig. 1:**
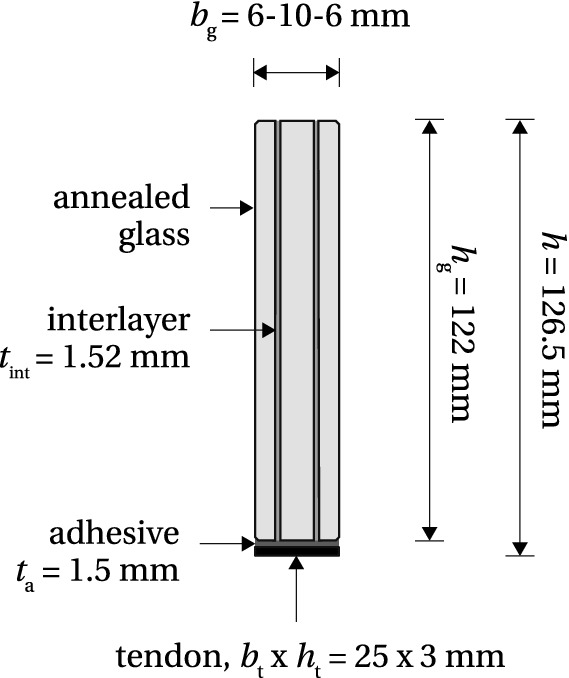
Schematic of the post-tensioned laminated glass beam cross-section with nominal dimensions

Present study focuses on the effectiveness of post-tension-ing in enhancing the fracture resistance of glass beams which depends on the level of compressive pre-stress introduced at the glass edge surface that will in service be exposed to tensile stresses induced by bending. The maximum pre-load that can be applied in a post-tensioned glass beam system, yielding maximum compressive pre-stress, is limited by a number of failure mechanisms which might occur during post-tensioning. This paper investigates the post-tensioning of laminated glass beams with an adhesively bonded flat stainless steel tendon placed along the bottom glass edge (Fig. 1). The tendon is first pre-tensioned by an external mechanism and subsequently adhesively bonded to the glass. The release of the pre-load set-up after the curing of the adhesive induces a compressive pre-stress and a hogging bending moment into the glass beam^1^. Failure mechanisms which may occur at this stage are the following: (1) rupture of the tendon, (2) glass fracture in tension^2^ due to the eccentricity of the pre-load, i.e. the hogging bending moment, (3) adhesive failure and (4) glass fracture caused by stress peaks in the load introduction zone at beam ends.

*The rupture of the steel tendon* is prevented by limiting the allowable stress induced by post-tensioning. In the related field of conventional prestressing steels applied in concrete structures, the maximum allowable stress is restricted to 75% of the characteristic tensile strength, or 85% of 0.1% proof stress (EN 1992-1-1 2004), in order to limit the loss of pre-load due to stress relaxation of steel under constant strain. Losses due to relaxation of prestressing steel are normally based on the value $\rho _{1000}$, the percentage relaxation loss at 1000 hours after tensioning at a mean temperature of $20\,^{\circ }\hbox {C}$, for an initial stress equal to 70% of the actual tensile strength of the prestressing steel samples prEN (2000). Stainless steel, which is not commonly applied for prestressing, exhibits relaxation in the same order of magnitude as conventional prestressing steels, with $\rho _{1000} < 8\%$ (Alonso et al. 2010), demonstrating by analogy that similar stress limitations may apply.

*Glass fracture* at the top glass edge is avoided by limiting the tensile stresses induced by the eccentrically applied pre-load. Maximum tensile stress at mid-span, for the initial pre-load level *P*, can be assessed from the following expression, assuming full composite action in the steel-glass section

1 $$\begin{aligned} \sigma _{\text {g,t,}P} = \frac{P}{A_{\text {eq}}} + \frac{Pe}{I_{\text {c}}} z_{\text {g,t}} \le f_{\text {g,d}} \end{aligned}$$

where $A_{\text {eq}}$ is the equivalent cross-sectional area of the beam, *e* is the eccentricity of the applied pre-load *P* from the neutral axis, $I_{\text {c}}$ is the moment of inertia of the composite section, and $z_{\text {g,t}}$ is the distance of the top glass edge from the neutral axis. Equivalent cross-sectional area is defined as

2 $$\begin{aligned} A_{\text {eq}} = \sum b_{i} h_{i} {E_{i}}/{E_{\text {g}}} \end{aligned}$$

where $b_{i}$, $h_{i}$, $E_{i}$ represent the width, height and Young’s modulus of the considered component of the section, and $E_{\text {g}}$ is the Young’s modulus of glass. The position of the neutral axis, in reference to the top edge of the beam, can be determined from the following expression

3 $$\begin{aligned} z_{\text {t}} = \frac{\sum b_{i} h_{i} z_{i \text {,t}} E_{i} / E_{\text {g}}}{\sum b_{i} h_{i} E_{i} / E_{\text {g}}} \end{aligned}$$

where $z_{i \text {,t}}$ is the distance from the centroid of the considered component to the top beam edge. The inertia of the composite section is calculated according to Eq. (4), following the Steiner’s rule

4 $$\begin{aligned} I_{\text {c}} = \sum \left( \frac{b_{i} h^{3}_{i}}{12} \frac{E_{i}}{E_{\text {g}}} + b_{i} h_{i} z^{2}_{i} \frac{E_{i}}{E_{\text {g}}} \right) \end{aligned}$$

where $z_{i}$ determines the distance of the centroid of a component to the neutral axis. The contribution of the interlayer foils in the calculation of the equivalent cross-sectional area and moment of inertia of a laminated glass beam can be neglected due to its several orders of magnitude lower Young’s modulus, relative to the other components of the section.

**Fig. 2:**
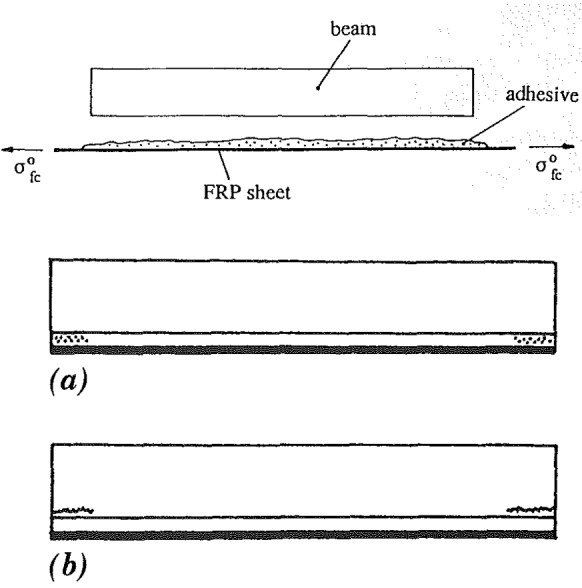
Concrete beams with external pretensioned FRP sheets; failure in the anchorage zone: **a** adhesive shear strength < beam shear strength, **b** adhesive shear strength > beam shear strength (Triantafillou and Deskovic 1991)

At the release of the pre-load from the post-tensioning set-up, *load introduction failure* may occur in the adhesive, the glass or at the tendon-adhesive/adhesive-glass interface, depending on the relative shear strength of the components of the load transfer. When the pre-load is too high, failure of the beam will occur at both beam ends due to high shear stresses which develop as the load is introduced from the tendon through the adhesive and into the glass. The transparent nature of glass limits the use of special anchorage which would provide vertical confinement in order to avoid this type of failure; thus, the design of the end zones requires special attention.

An analytical model which describes the short-term mechanical behaviour of post-tensioning through bonded tendons is presented in Sect. 2. It is based on the model developed by Triantafillou and Deskovic (1991) for concrete beams externally post-tensioned through fiber-reinforced plastic (FRP) composite sheets bonded in the tensile zone of a structural element (Fig. 2). The model allows for determination of the maximum allowable pre-load, for two failure scenarios: (1) *cohesive failure of the adhesive* (within the bulk material) in a system with superior glass shear strength, (2) *glass fracture* in a system with superior shear strength of the adhesive. *Adhesive* strength on both substrates is considered sufficiently high to avoid failure at the interface, assuming appropriate surface preparation prior to bonding (steel surface may be roughened with sand-paper, followed by thorough cleaning, of both steel and glass, with isopropyl alcohol; glass primer is applied on the glass surface to improve adhesion). In Sect. 3, the analytical model is used for the calculation of the allowable pre-load for a beam specimen applied in a wider experimental study on the bending behaviour of post-tensioned glass beams (Cupać et al. 2021); the results of the analytical model are further verified with a numerical model of the investigated beam system. Finally, the model is applied in a parametric study, presented in Sect. 4, in order to determine the influence of certain geometric beam parameters and adhesive properties on the effectiveness of the post-tensioning. The results are discussed in Sect. 5, with conclusions given in Sect. 6.

## Analytical model of pre-load introduction

**Fig. 3:**
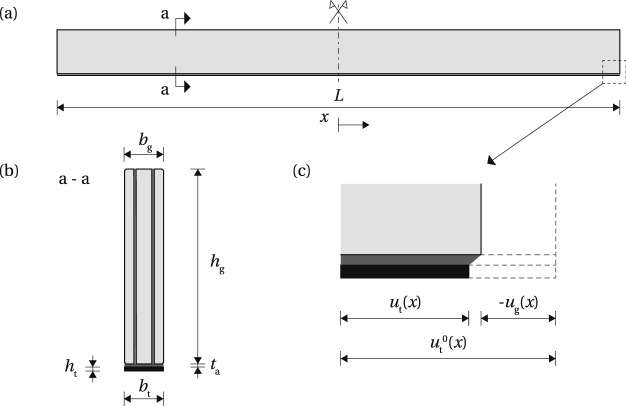
Components of the model of the post-tensioned beam system; **a** longitudinal and **b** cross-section, **c** axial deformations at the beam end upon release of the pre-load; adapted from Triantafillou and Deskovic (1991)

The glass beam shown in Fig. 3 has a length *L*, height $h_{\text {g}}$ and width^3^$b_{\text {g}}$. Pre-stressed tendon is bonded at the bottom glass edge; the height and width of the tendon is $h_{\text {t}}$ and $b_{\text {t}}$, respectively. The adhesive thickness is $t_{\text {a}}$. Young’s modulus of the glass beam is $E_{\text {g}}$, Young’s modulus of the tendon is $E_{\text {t}}$, and the shear modulus of the adhesive is $G_{\text {a}}$. The tendon is initially pre-stressed to a stress level of $\sigma ^{0}_{\text {t}}$. Upon release of the tendon from the post-tensioning set-up, the stress at a distance *x* from the beam mid-length drops to $\sigma _{\text {t}} (x)$. The pre-stress is transferred into the glass through the adhesive layer, resulting in a shear stress $\tau (x)$ at the interface, and a compressive stress $\sigma _{\text {g,b}} (x)$ at the bottom glass edge. The shear stress distribution is considered uniform across the adhesive thickness; peeling stresses are assumed to be negligible for the investigated tendon thickness, i.e. not causing delamination. Given the relatively small thickness of the adhesive and the tendon, these simplifying assumptions are considered acceptable for a derivation of a theoretical solution which aims to provide initial understanding of the mechanics of load-introduction in a post-tensioned glass beam system. The release of pre-stress is accompanied by a displacement in the beam components, shown in Fig. 3c (rotation, i.e. peeling, is here neglected for simplicity). The initial state, just before the release, is marked with a dashed line; the solid lines indicate the state of displacement just after the release. The initial extension of the tendon at a distance *x* equals $u^{0}_{\text {t}} (x)$. The release of the pre-stress causes elastic shortening of the glass beam, which equals $-u_{\text {g}} (x)$ at the bottom glass edge, while the deformation of the tendon drops to $u_{\text {t}} (x)$.

Assuming linear-elastic material behaviour, shear strain $\gamma$ and shear stress $\tau$ can be defined as follows

5 $$\begin{aligned} \gamma= & {} \frac{u^{0}_{\text {t}} - u_{\text {t}} + u_{\text {g}}}{t_{\text {a}}} \end{aligned}$$

6 $$\begin{aligned} \tau= & {} \frac{G_{\text {a}}}{t_{\text {a}}} (u^{0}_{\text {t}} - u_{\text {t}} + u_{\text {g}}) \end{aligned}$$

Equation (6) differentiated with respect to *x* equals

7 $$\begin{aligned} \begin{aligned} \frac{d \tau }{d x}&= \frac{G_{\text {a}}}{t_{\text {a}}} \left( \frac{d u^{0}_{\text {t}}}{dx} - \frac{d u_{\text {t}}}{dx} + \frac{d u_{\text {g}}}{dx} \right) \\&= \frac{G_{\text {a}}}{t_{\text {a}}} \left( \frac{\sigma ^{0}_{\text {t}}}{E_{\text {t}}} - \frac{\sigma _{\text {t}}}{E_{\text {t}}} + \frac{\sigma _{\text {g,b}}}{E_{\text {g}}} \right) \end{aligned} \end{aligned}$$

Compressive pre-stress at the bottom glass edge, $\sigma _{\text {g,b}}$, is assumed uniform across the width of the glass beam, for the sake of simplicity. It can be expressed in terms of the tensile stress in the tendon, $\sigma _{\text {t}}$, through the following equation

8 $$\begin{aligned} \begin{aligned} \sigma _{\text {g,b}}&= -\frac{b_{\text {t}} h_{\text {t}} \sigma _{\text {t}}}{A_{\text {g}}} - \frac{b_{\text {t}} h_{\text {t}} \sigma _{\text {t}} e z_{\text {g,b}}}{I_{\text {g}}} \\&= -\left( \frac{b_{\text {t}} h_{\text {t}}}{A_{\text {g}}} + \frac{b_{\text {t}} h_{\text {t}} e z_{\text {g,b}}}{I_{\text {g}}} \right) \sigma _{\text {t}} \\&= - \alpha \sigma _{\text {t}} \end{aligned} \end{aligned}$$

where $A_{\text {g}}$ is the area and $I_{\text {g}}$ the moment of inertia of the glass beam, $z_{\text {g,b}}$ is the distance from the glass centroid to the bottom glass edge, *e* is the eccentricity of the force acting in the centroid of the tendon ($b_{\text {t}} h_{\text {t}} \sigma _{\text {t}}$) from the glass centroid, thus $e=z_{\text {g,b}} + t_{\text {a}} + h_{\text {t}}/2$, and

9 $$\begin{aligned} \alpha = \frac{b_{\text {t}} h_{\text {t}}}{A_{\text {g}}} + \frac{b_{\text {t}} h_{\text {t}} e z_{\text {g,b}}}{I_{\text {g}}} \end{aligned}$$

By substituting Eq. (8) into (7), the following is obtained

10 $$\begin{aligned} \frac{d \tau }{d x} = \frac{G_{\text {a}}}{t_{\text {a}}} \left[ \frac{\sigma ^{0}_{\text {t}}}{E_{\text {t}}} - \left( \frac{1}{E_{\text {t}}} + \frac{\alpha }{E_{\text {g}}} \right) \sigma _{\text {t}} \right] \end{aligned}$$

which, differentiated with respect to *x*, results in

11 $$\begin{aligned} \frac{d^{2} \tau }{d x^{2}} = - \frac{G_{\text {a}}}{t_{\text {a}}} \left( \frac{1}{E_{\text {t}}} + \frac{\alpha }{E_{\text {g}}} \right) \frac{d \sigma _{\text {t}}}{d x} \end{aligned}$$

The equilibrium of the tendon under tensile stress, $\sigma _{\text {t}}$, and shear stress, $\tau$, at the interface with the adhesive, can be expressed as

12 $$\begin{aligned} h_{\text {t}} \frac{d \sigma _{\text {t}}}{d x} = - \tau \end{aligned}$$

Equation (11) then becomes a second order linear homogeneous equation

13 $$\begin{aligned} \frac{d^{2} \tau }{d x^{2}} = \frac{G_{\text {a}}}{h_{\text {t}} t_{\text {a}}} \left( \frac{1}{E_{\text {t}}} + \frac{\alpha }{E_{\text {g}}} \right) \tau = \omega ^{2} \tau \end{aligned}$$

where

14 $$\begin{aligned} \omega ^{2} = \frac{G_{\text {a}}}{h_{\text {t}} t_{\text {a}}} \left( \frac{1}{E_{\text {t}}} + \frac{\alpha }{E_{\text {g}}} \right) \end{aligned}$$

A general solution of Eq. (13) is of the form

15 $$\begin{aligned} \tau = C_{1} e^{\omega x} + C_{2} e^{- \omega x} \end{aligned}$$

The coefficients $C_{1}$ and $C_{2}$ can be determined from the boundary conditions, which depend on the considered failure mechanism. The failure is governed by the shear strength of the glass or the adhesive, whichever is lower. The following subsections provide the solution for the allowable level of initial pre-stress in the tendon for the two failure mechanisms.

**Fig. 4:**
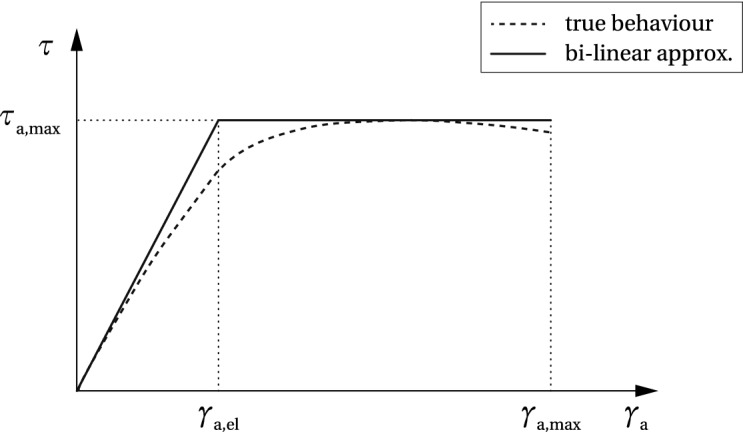
Shear stress-shear strain curve for a thermoset structural adhesive; dashed line—true behaviour based on the tests on Araldite^®^ 2047 from Nhamoinesu (2015); solid line—bilinear approximation of the stress-strain curve

**Fig. 5:**
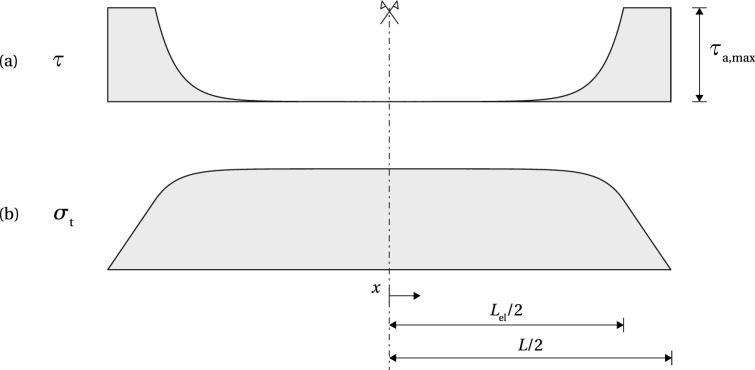
Stress distribution along the beam at the limit of the adhesive shear capacity; **a** shear stress at the interface; **b** tensile stress in the tendon; adapted from Triantafillou and Deskovic (1991)

### Allowable pre-load governed by the shear strength of the adhesive (model AF)

The shear stress-shear strain relationship for a thermoset structural adhesive is schematically shown in Fig. 4. The dashed line shows the behaviour of a two component meth-acrylate adhesive Araldite^®^ 2047 in a single lap shear test, adopted from (Nhamoinesu 2015). In the current model, the true behaviour is approximated by a bilinear curve (solid line), describing two characteristic behaviour modes: the initial linear-elastic response up to the strain level of $\gamma _{\text {a,el}}$, followed by the perfectly plastic path leading to failure once the strain limit $\gamma _{\text {a,max}}$ is reached. The shear strength equals $\tau _{\text {a,max}}$.

The release of the pre-load induces high shear stresses at beam ends. Figure 5a shows the stress distribution along the beam at the limit of the shear capacity of the adhesive (note that $x=0$ is located at beam mid-span): in the elastic range $0 \le x \le L_{\text {el}}/2$, the stress distribution is described by Eq. (15); for $L_{\text {el}}/2 \le x \le L/2$, the shear stress equals $\tau _{\text {a,max}}$. The corresponding shear strain equals $\gamma _{\text {a,el}}$ at $x = L_{\text {el}}/2$, and $\gamma _{\text {a,max}}$ at $x = L/2$. The coefficients $C_{1}$ and $C_{2}$ can be determined from the following boundary conditions

16 $$\begin{aligned}&\tau (x = 0) = 0 \end{aligned}$$

17 $$\begin{aligned}&\gamma (x = L_{\text {el}}/2) = \tau (x = L_{\text {el}}/2) / G_{\text {a}} = \gamma _{\text {a,el}} \end{aligned}$$

resulting in

18 $$\begin{aligned} C_{1} = \frac{\gamma _{\text {a,el}} G_{\text {a}}}{2 \sinh (\omega L_{\text {el}}/2)} \; \; \text {and} \; \; C_{2} = -\frac{\gamma _{\text {a,el}} G_{\text {a}}}{2 \sinh (\omega L_{\text {el}}/2)} \end{aligned}$$

**Fig. 6:**
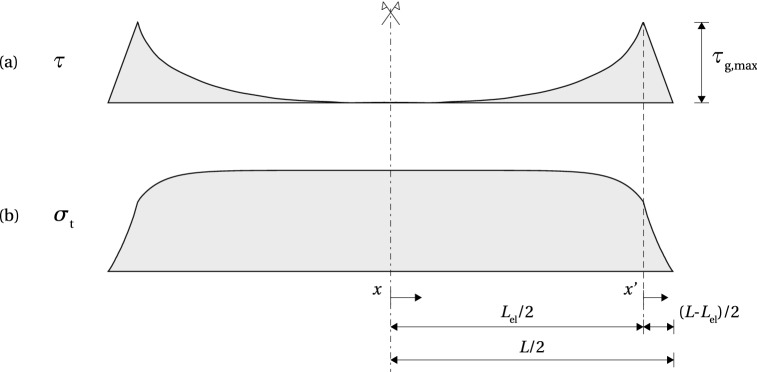
Stress distribution along the beam at the limit of the glass shear capacity; **a** shear stress at the interface; **b** tensile stress in the tendon; adapted from (Triantafillou and Deskovic 1991)

By substituting (18) into (15), the following expression is obtained for the shear stress distribution in the elastic zone

19 $$\begin{aligned} \tau = \frac{\gamma _{\text {a,el}} G_{\text {a}}}{\sinh (\omega L_{\text {el}}/2)} \sinh (\omega x), \qquad 0 \le x \le L_{\text {el}}/2 \end{aligned}$$

In the plastic zone, $L_{\text {el}}/2 \le 0 \le L/2$, the shear stress is constant; however, the shear strain is assumed to follow the same distribution as in the elastic zone, hence

20 $$\begin{aligned} \gamma = \frac{\gamma _{\text {a,el}}}{\sinh (\omega L_{\text {el}}/2)} \sinh (\omega x), \qquad 0 \le x \le L/2 \end{aligned}$$

The length of the elastic zone, $L_{\text {el}}$, follows from the condition $\gamma (x = L/2) = \gamma _{\text {a,max}}$

21 $$\begin{aligned}&L_{\text {el}} = \frac{2 \ln \left( \dfrac{\beta + \sqrt{\beta ^{2} +4}}{2} \right) }{\omega }, \quad \text {where} \end{aligned}$$

22 $$\begin{aligned}&\beta = \frac{2 \gamma _{\text {a,el}}}{\gamma _{\text {a,max}}} \sinh (\omega L/2) \end{aligned}$$

The tensile stress distribution along the tendon, in the elastic zone, can be obtained starting from Eq. (10); $\tfrac{d \tau }{d x}$ can be substituted with a derivative of (19) with respect to *x*

23 $$\begin{aligned} \sigma _{\text {t}} = \frac{\sigma ^{0}_{\text {t}} - \dfrac{E_{\text {t}} t_{\text {a}} \omega \gamma _{\text {a,el}}}{\sinh (\omega L_{\text {el}}/2)} \cosh (\omega x)}{1 + \alpha \dfrac{E_{\text {t}}}{E_{\text {g}}}}, \qquad 0 \le x \le L_{\text {el}}/2\nonumber \\ \end{aligned}$$

In the plastic zone, the tensile stress linearly drops from $\sigma _{\text {t}} (x = L_{\text {el}}/2)$ to zero at $x = L/2$ (Fig. 5b). The condition of slope continuity of $\sigma _{\text {t}}$ at $x = L_{\text {el}}/2$ can be written as

24 $$\begin{aligned} \left. \frac{d\sigma _{\text {t}}}{dx}\right| _{x=L_{\text {el}}/2} = \frac{\left. \sigma _{\text {t}}\right| _{x=L_{\text {el}}/2}}{\dfrac{L-L_{\text {el}}}{2}} \end{aligned}$$

Solving Eq. (24) for $\sigma ^{0}_{\text {t}}$ results in the expression for the initial pre-stress level that will just cause failure in the adhesive upon release from the post-tensioning rig

25 $$\begin{aligned} \sigma ^{0}_{\text {t}} = E_{\text {t}} t_{\text {a}} \omega \gamma _{\text {a,el}} \left[ \coth (\omega L_{\text {el}}/2) + \frac{\omega (L-L_{\text {el}})}{2} \right] \end{aligned}$$

From $\sigma _{\text {t}}$, which can now be obtained from (23), the corresponding compressive stress at the lower glass edge, $\sigma _{\text {g,b}}$, can be calculated applying (8).

### Allowable pre-load governed by the shear strength of glass (model GF)

When applying structural adhesives with high shear stiffness and shear strength, fracture in glass may occur at the release of the pre-load in the set-up, or with some delay. Once the shear stress at the beam end reaches the level of glass resistance in shear (< adhesive shear strength), a crack is initiated at the lower glass edge, which results in a drop in shear stress towards the beam end.

**Fig. 7:**
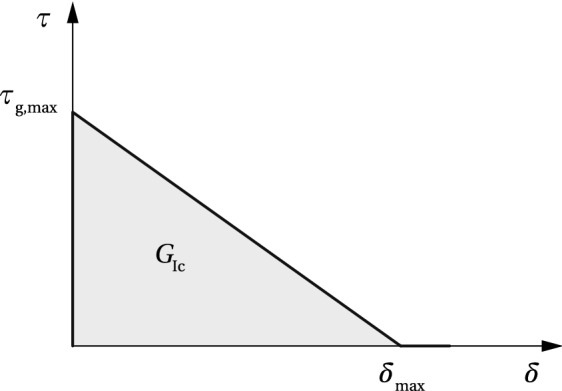
Simplified model for shear stress-slip relationship in glass

**Fig. 8:**
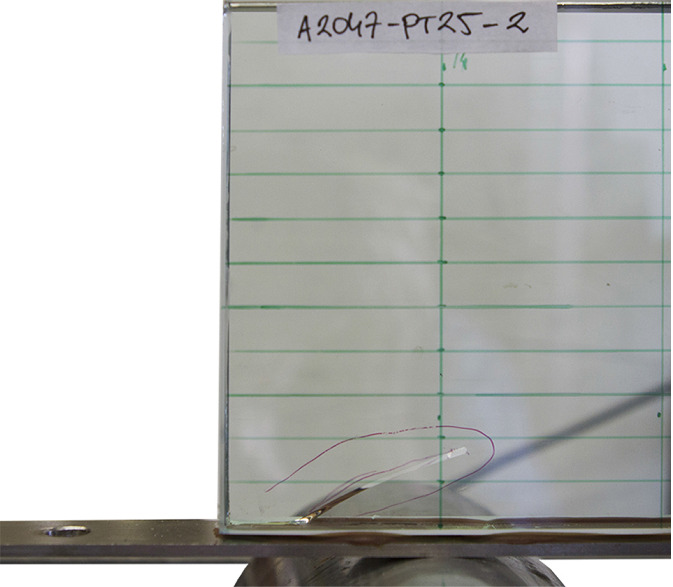
Shear crack at the release from the post-tensioning set-up

The distribution of the shear stress is schematically shown in Fig. 6a. The elastic zone, $0 \le x \le L_{\text {el}}/2$, is described by Eq. (15); in the non-linear zone, $0 \le x' \le (L-L_{\text {el}})/2$, the fracturing behaviour is described by a softening law which relates the shear stress at the interface ($\tau$) with a relative slip between the substrates ($\delta$). In the lack of an existing model for this type of failure in glass, an analogy with the softening of concrete in shear is assumed. A non-linear softening law is approximated with a simplified linearly descending $\tau - \delta$ model (Yuan et al. 2001), shown in Fig. 7. Once the fracture at the interface is initiated at $\tau _{\text {g,max}}$, the stress linearly reduces with the increase of slip, reaching zero when the value of slip exceeds $\delta _{\text {max}}$. The area below the curve presents the fracture energy in mode I, $G_{\text {Ic}}$, i.e. the energy dissipated in the formation of new fracture surfaces^4^, in case of brittle materials. It should be noted that, unlike in concrete, where the cracks propagate in mode II (*sliding*) in a layer above the tendon, parallel with the interface (Triantafillou and Deskovic 1991), pure mode I (*opening*) is assumed the governing mode for crack propagation in glass. The observed cracking at the release of the pre-load (Fig. 8) shows an opening crack, which propagates perpendicularly to the direction of the maximum principal stresses (Sect. 3.2).

Coefficients $C_{1}$ and $C_{2}$ in Eq. (15) can be determined from the boundary conditions

26 $$\begin{aligned}&\tau (x = 0) = 0 \end{aligned}$$

27 $$\begin{aligned}&\tau (x = L_{\text {el}}/2) = \tau _{\text {g,max}} \end{aligned}$$

resulting in the following expression for the shear stress distribution in the elastic zone

28 $$\begin{aligned} \tau = \frac{\tau _{\text {g,max}}}{\sinh (\omega L_{\text {el}}/2)} \sinh (\omega x), \qquad 0 \le x \le L_{\text {el}}/2 \end{aligned}$$

Similarly to (23), the distribution of the tensile stress in the tendon can be determined from (10), substituting $\tfrac{d \tau }{d x}$ with a derivative of (28)

29 $$\begin{aligned} \sigma _{\text {t}} = \frac{\sigma ^{0}_{\text {t}} - \dfrac{E_{\text {t}} t_{\text {a}} \omega \tau _{\text {g,max}}}{ G_{\text {a}} \sinh (\omega L_{\text {el}}/2)} \cosh (\omega x)}{1 + \alpha \dfrac{E_{\text {t}}}{E_{\text {g}}}}, \qquad 0 \le x \le L_{\text {el}}/2 \end{aligned}$$

The shear slip $\delta$ can be determined from the relative displacement of the substrates at the release of the pre-load. Assuming a fully rigid glass-adhesive system in the non-linear zone, the slip at distance $x'$ results only from the straining of the tendon

30 $$\begin{aligned} \frac{d(u^{0}_{\text {t}} - u_{\text {t}})}{dx'} = \frac{du^{0}_{\text {t}}}{dx'} - \frac{du_{\text {t}}}{dx'} = \varepsilon ^{0}_{\text {t}} - \varepsilon _{\text {t}} \end{aligned}$$

The shear slip follows from

31 $$\begin{aligned} \delta (x') = \int ^{x'}_{0} (\varepsilon ^{0}_{\text {t}} - \varepsilon _{\text {t}}) dx' = \frac{\sigma ^{0}_{\text {t}}}{E_{\text {t}}} x' - \int ^{x'}_{0} \frac{\sigma _{\text {t}}}{E_{\text {t}}} dx' \end{aligned}$$

Linear approximation of the shear distribution in the non-linear zone can be written as

32 $$\begin{aligned} \tau = \tau _{\text {g,max}} \left( 1 - \frac{x'}{\dfrac{L-L_{\text {el}}}{2}} \right) , \qquad 0 \le x' \le (L-L_{\text {el}})/2 \end{aligned}$$

The equilibrium of the tendon under tensile stress and interface shear stress, taken as a triangle, at a distance $x'$ in the non-linear zone, equals

33 $$\begin{aligned} h_{\text {t}} \sigma _{\text {t}} = \frac{1}{2} \tau \left( \frac{L-L_{\text {el}}}{2} - x'\right) \end{aligned}$$

The combination of (32) and (33), solved for $\sigma _{\text {t}}$, gives the expression for the distribution of the tensile stress in the tendon

34 $$\begin{aligned} \sigma _{\text {t}} = \frac{\tau _{\text {g,max}}}{h_{\text {t}} (L-L_{\text {el}})} \left( \frac{L-L_{\text {el}}}{2} - x' \right) ^{2}, \qquad 0 \le x' \le (L-L_{\text {el}})/2\nonumber \\ \end{aligned}$$

Substituting (34) into (31), after integration gives the following

35 $$\begin{aligned} \begin{aligned} \delta (x') =&\frac{\sigma ^{0}_{\text {t}}}{E_{\text {t}}} x' - \frac{\tau _{\text {g,max}} (L-L_{\text {el}})}{4 E_{\text {t}} h_{\text {t}}} x' + \frac{\tau _{\text {g,max}}}{2 E_{\text {t}} h_{\text {t}}} (x')^{2} \\&- \frac{\tau _{\text {g,max}}}{3 E_{\text {t}} h_{\text {t}}(L-L_{\text {el}})} (x')^{3} + C \end{aligned} \end{aligned}$$

For the condition $\delta (x'=0)=0$, (35) results in $C=0$. For $\delta (x'=(L-L_{\text {el}})/2)=\delta _{\text {max}}$, Eq. (35) becomes

36 $$\begin{aligned} \delta _{\text {max}} = \frac{\sigma ^{0}_{\text {t}} (L-L_{\text {el}})}{2 E_{\text {t}}} - \frac{\tau _{\text {g,max}}(L-L_{\text {el}})^{2}}{24 E_{\text {t}}} \end{aligned}$$

The tensile stress in the tendon following from Eq. (29) for $x=L_{\text {el}}/2$ should be equal to that calculated from (34) for $x'=0$. This condition can be written as

37 $$\begin{aligned} \frac{\sigma ^{0}_{\text {t}} - \dfrac{E_{\text {t}} t_{\text {a}} \omega \tau _{\text {g,max}}}{ G_{\text {a}}} \coth (\omega L_{\text {el}}/2)}{1 + \alpha \dfrac{E_{\text {t}}}{E_{\text {g}}}} = \frac{(L-L_{\text {el}}) \tau _{\text {g,max}}}{4 h_{\text {t}}} \end{aligned}$$

Knowing $\tau _{\text {g,max}}$ and $\delta _{\text {max}}$, equations (36) and (37) can be solved for the two remaining unknowns, the length of the elastic zone, $L_{\text {el}}$, and the initial pre-stress in the tendon, $\sigma ^{0}_{\text {t}}$, that will just initiate fracture in glass upon release. $L_{\text {el}}$ can be substituted in (29) and (34) to obtain the distribution of the tensile stress in the tendon in the linear and non-linear zone, respectively (Fig. 6b). Finally, the compressive pre-stress at the bottom glass edge, $\sigma _{\text {g,b}}$, can be obtained from (8).

**Fig. 9:**
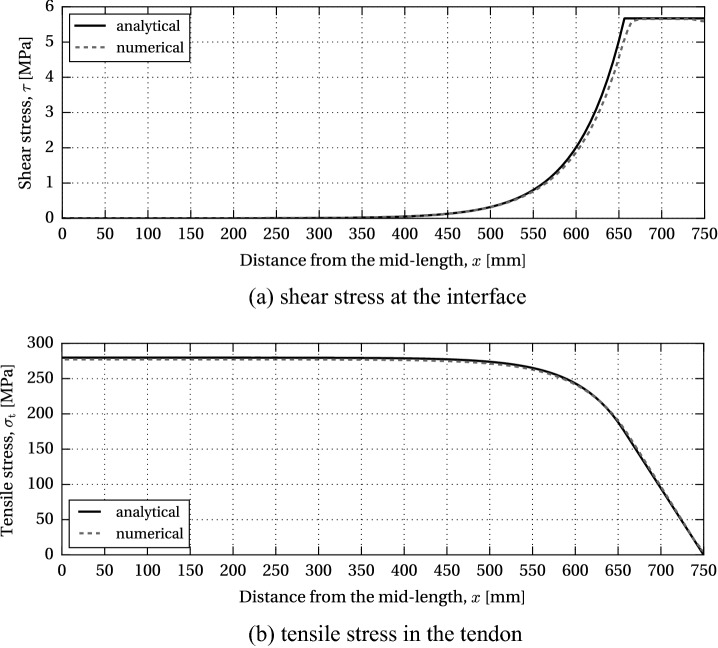
Shear and tensile stress distribution at the limit of the adhesive shear capacity; comparison of the analytical and numerical model

## Numerical verification of the model

The analytical model was first applied for the calculation of the allowable pre-load and stress distribution of a glass beam post-tensioned through an adhesively bonded tendon placed along the bottom glass edge (Fig. 1). In order to verify the analytical results, a numerical 2D model of the beam was implemented in a finite element (FE) software Abaqus^5^, version 6.12-3.

The beam comprises a triple-laminated annealed glass section ($6+10+6$ mm) with a height of 122 mm and a length of 1500 mm. The pre-stress is applied via stainless steel tendon $25\times 3$ mm, grade EN 1.4301 (EN 10088-1 2005), and transferred into the glass through 1.5 mm thick adhesive bond. The applied Young’s modulus of glass, $E_{\text {g}}$, and the tendon, $E_{\text {t}}$, equal 70 GPa (EN 572-1 2004) and 180 GPa [based on uniaxial tensile tests reported in Cupać (2017)], respectively. Two types of adhesives were considered, in order to simulate the two failure modes represented by the models AF and GF. Adhesive properties are further detailed in the following sections where the two models are investigated separately.

### Model AF

For the model AF, governed by the adhesive strength, Aral-dite^®^ 2047-1 was selected as the reference adhesive. The parameters defining the bilinear shear stress-shear strain cur-ve were assessed based on the experimental results reported in Nhamoinesu (2015); the following values were adopted in the model: $G_{\text {a}} = 211$ MPa, $\gamma _{\text {a,el}}=2.69\%$, $\gamma _{\text {a,max}}=15\%$, $\tau _{\text {a,max}}=5.67$ MPa. For the given beam properties, the initial pre-stress level that will just cause failure in the adhesive upon release, $\sigma ^{0}_{\text {t}}$, amounts to 363.24 MPa, i.e. the initial pre-load $P=27.24$ kN. The corresponding compressive pre-stress at the bottom glass edge, $\sigma _{\text {g,b}}$, equals $-32.42$ MPa. Figure 9 shows the distribution of the shear stress in the adhesive, $\tau$, and the tensile stress in the tendon, $\sigma _{\text {t}}$, for $0 \le x \le L/2$, resulting from the release of the initial pre-load *P* (solid curves). The length of the elastic zone, $L_{\text {el}}$, equals 1313 mm, i.e. the adhesive yielding at the beam ends occurs over the initial 94 mm.

**Fig. 10:**
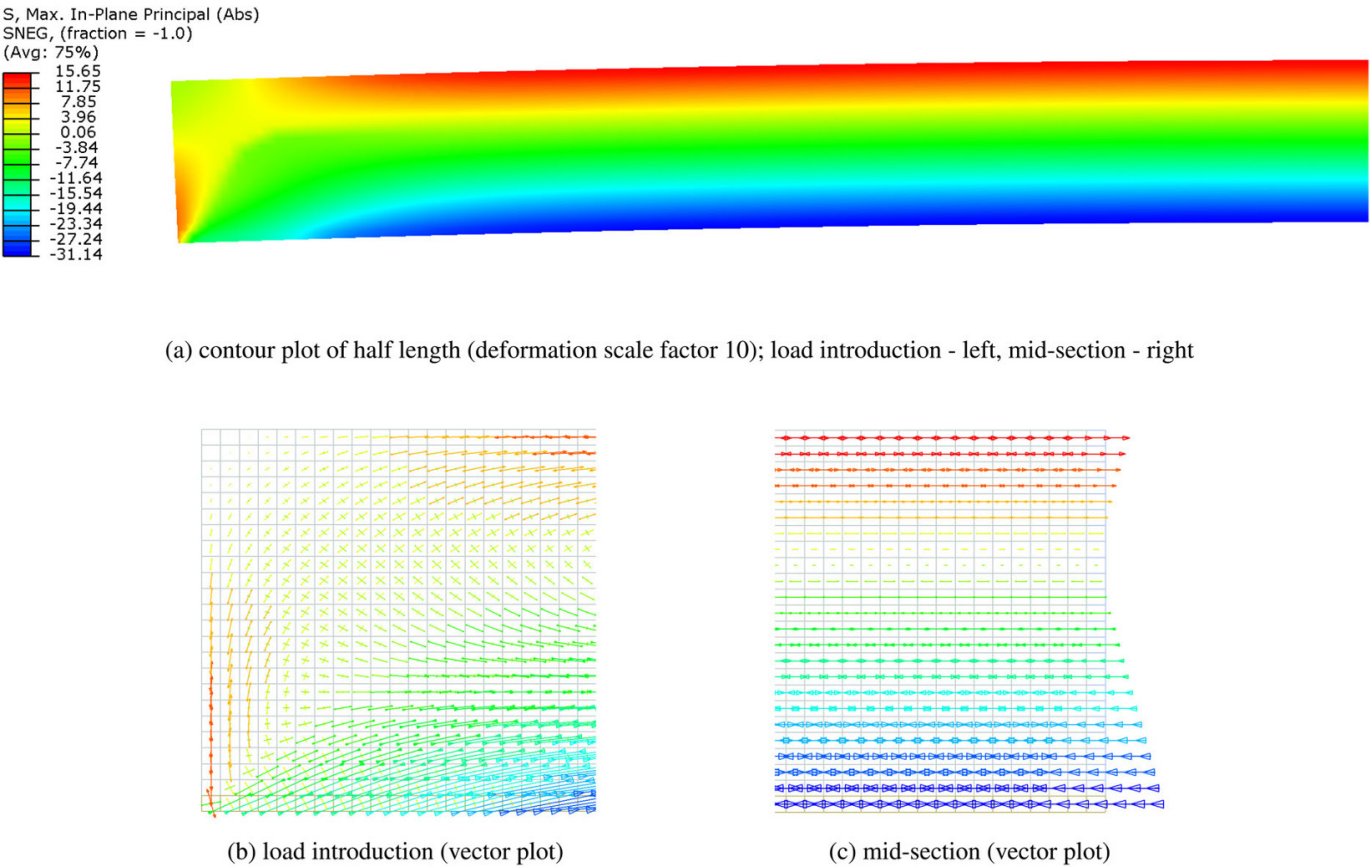
Distribution of principal stresses in the glass (half length) at the limit of the adhesive shear capacity (FEM results)

In the numerical model, only half of the beam length *L* was considered, with symmetry restraint at the mid-section nodes reproducing the effective boundary conditions. The beam components - glass, tendon and adhesive—were represented with 4-node monolithic shell elements with reduced integration (S4R). A regular mesh pattern was applied, with element size of 5 mm along the beam length. Glass height was divided in 24 elements (element size $\sim 5\times 5$ mm), three elements were applied across the thickness of the adhesive ($0.5\times 5$ mm), and one element over the height of the tendon ($3\times 5$ mm), resulting in a total of 4200 elements. A rigid constraint (*tie*) was used at the tendon-adhesive and adhesive-glass interface. Material properties equivalent to those applied in the analytical model AF were implemented in the numerical simulation. The initial pre-stress level, $\sigma ^{0}_{\text {t}}=363.24$ MPa, obtained through the analytical solution, was applied on the tendon as a pre-defined field (*mechanical/ stress*) in the initial step of the simulation. A geometrically non-linear, static incremental computation was performed in Abaqus/Standard. The resulting stress distribution in the adhesive and the tendon is plotted in Fig. 9 (dashed curves). The stress data represents the averaged nodal values extrapolated from the integration points of the connecting elements. The stress plots resulting from the analytical and numerical simulation demonstrate a good correlation in the computation of both shear stresses in the adhesive layer and tensile stresses in the tendon. The distribution of the principal stresses in glass is shown in Fig. 10. The load-introduction zone (Fig. 10b) is subjected to a complex stress state, which tends to a linear stress-distribution over the beam height, as the pre-stress is gradually introduced into the glass. In the mid-section, the stress varies linearly from tension at the top edge to compression at the bottom (Fig. 10c). The maximum value of compressive pre-stress in the FE model, extrapolated to the bottom glass edge, equals $-32.16$ MPa, which closely corresponds to the value of $-32.42$ MPa, obtained analytically.

**Fig. 11:**
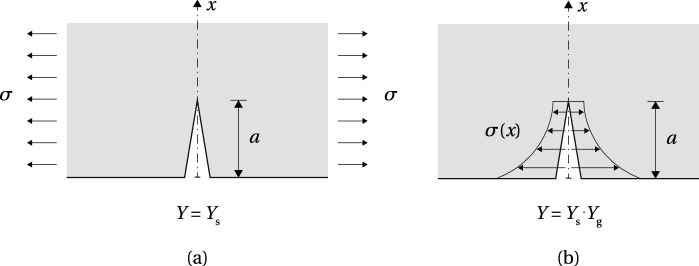
Surface crack in a semi-infinite body with a **a** uniform and **b** non-uniform stress distribution along the crack depth

### Model GF

For the model GF, epoxy adhesive 3M™ Scotch-Weld™ DP490 was chosen as the reference adhesive due to its relatively high shear modulus and shear strength, compared to other adhesives, such as Araldite^®^ 2047. The shear modulus $G_{\text {a}}$ equals 239 MPa (Nhamoinesu 2015), the shear strength $\tau _{\text {a,max}}=30.2$ MPa at $23\,^{\circ }\hbox {C}$, according to the manufacturer’s data sheet (3M 1996).

DP490 was applied for the post-tensioning of glass beams with the same nominal parameters (Fig. 1) in the scope of a master’s thesis (Cokragan 2015); glass failure was consistently observed at beam ends at the release of a 15 kN pre-load. In order to determine the maximum shear stress at the interface which initiated glass fracture, $\tau _{\text {g,max}}$, i.e. the shear resistance of glass, the release of the pre-load was simulated in a 2D numerical model in the present study.

The results of the numerical model were further applied in the calculation of the stress intensity factor (SIF), $K_{\text {I}}$, based on the approach proposed by Albrecht and Yamada (1977). The procedure is based on the *linear superposition principle* (Broek 1986) used in linear elastic fracture mechanics (LEFM) calculations to derive the SIF from an uncracked FE model, with the assumption that the cracking does not significantly influence the global stiffness of the component. The correction factor *Y* is divided in two parts, $Y=Y_{\text {s}} Y_{\text {g}}$, where $Y_{\text {s}}$ accounts for the crack shape and the proximity of boundaries in a cracked body with a uniform stress distribution, and $Y_{\text {g}}$ is the correction factor for the local stress gradient due to the geometry of the modelled structural detail (Fig. 11). The expression for the SIF therefore equals

38 $$\begin{aligned} K_{\text {I}} = Y_{\text {s}} (Y_{\text {g}} \sigma \sqrt{\pi a}) = Y_{\text {s}} K'_{\text {I}} \end{aligned}$$

where $Y_{\text {s}}=1.12$ for a shallow surface crack in a semi-infinite solid (Irwin 1962). The value of the SIF $K'_{\text {I}}$, which contains the correction factor $Y_{\text {g}}$, can be determined in two steps by (1) computing the stresses in an uncracked model along a line where the anticipated crack will be inserted and (2) integrating the normal stresses along the same line, for a given crack depth, by applying the following expression

39 $$\begin{aligned} K'_{\text {I}} = \sqrt{\pi a} \dfrac{2}{\pi } \int ^{a}_{0} \dfrac{\sigma (x)}{\sqrt{a^{2}-x^{2}}} dx \end{aligned}$$

where *a* is the crack depth, $\sigma (x)$ is the stress distribution along the anticipated crack path, and *x* is the location along the crack path. For discrete values of stress obtained from the FEM, (39) becomes

40 $$\begin{aligned} K'_{\text {I}} = \sqrt{\pi a} \dfrac{2}{\pi } \sum ^{n}_{i=1} \sigma _{i} \left( \text {arcsin} \dfrac{x_{i+1}}{a} - \text {arcsin} \dfrac{x_{i}}{a} \right) \end{aligned}$$

where $\sigma _{i}$ is the discrete stress normal to the crack path, applied over the element width from $x_{i}$ to $x_{i+1}$, and summed over the total number of elements along the crack depth *a*.

The numerical model applied for the simulation of pre-load introduction at the verge of adhesive failure was adapted by changing the material properties of the adhesive; DP490 was modelled as linear-elastic, with Young’s modulus $E=660$ MPa and Poisson’s ratio $\nu =0.38$ (Nhamoinesu 2015). The adhesive was applied with a 5 mm offset from the beam end, corresponding to the bonding layout applied in the experiments to avoid stressing the glass edge (5 mm long soft double-sided adhesive pads were applied between the glass and the tendon to prevent the spread of adhesive to the glass beam corner edge). The mesh was refined in the zone of the load-introduction, to allow for the computation of $K'_{\text {I}}$, with 0.003 mm elements over an area of $0.4\times 0.2$ mm. The element size was gradually increased towards the edges of the beam, to a maximum size of 5 mm, resulting in a total of 28648 elements. Initial pre-stress of 200 MPa was applied on the tendon elements, which corresponds to a 15 kN axial pre-load.

**Fig. 12:**
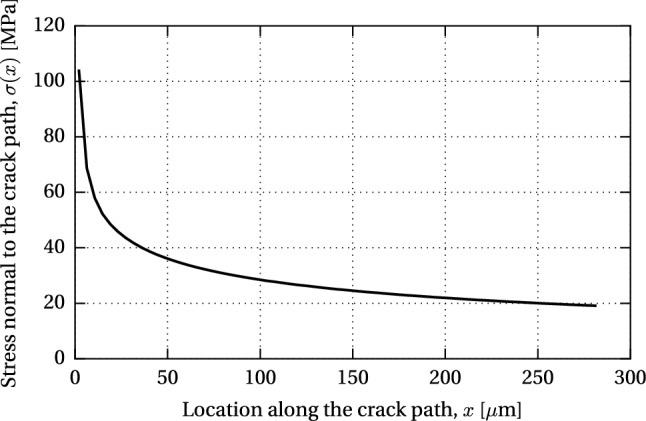
Stress gradient along the crack path perpendicular to the direction of the maximum principal stresses

**Fig. 13:**
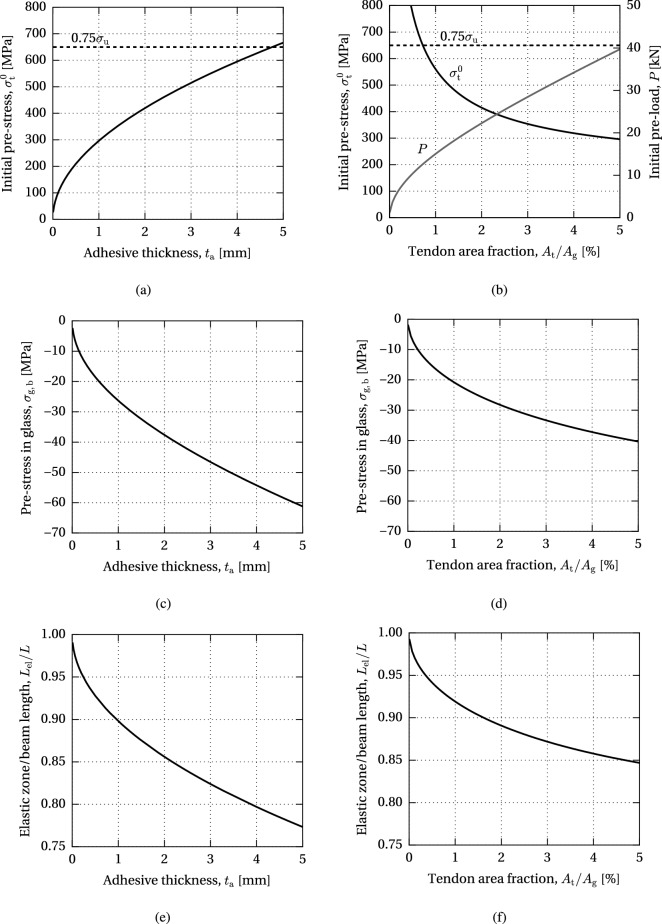
Variation of the allowable initial pre-stress level, resulting compressive pre-stress on the bottom glass edge and length of the elastic zone with respect to the adhesive thickness and tendon area fraction (model AF)

The maximum shear stress of 8.28 MPa, obtained from the numerical model, was adopted as the glass shear resistance, $\tau _{\text {g,max}}$, for this specific geometry and mechanism of load-introduction. For a known $\tau _{\text {g,max}}$, the maximum shear slip, $\delta _{\text {max}}$, can be derived from the simplified $\tau -\delta$ relationship shown in Fig. 7

41 $$\begin{aligned}&\delta _{\text {max}} = \dfrac{2G_{\text {Ic}}}{\tau _{\text {g,max}}} \end{aligned}$$

42 $$\begin{aligned}&G_{\text {Ic}} = \dfrac{K^{2}_{\text {Ic}} (1-\nu _{\text {g}}^{2})}{E_{\text {g}}} \end{aligned}$$

where fracture toughness $K_{\text {Ic}} = 0.75~\text {MPa}\sqrt{\text {m}}$ and Poisson’s ratio $\nu _{\text {g}} = 0.23$ (Haldimann et al. 2008). The resulting maximum shear slip equals $\delta _{\text {max}}=1.8$ µm.

The first path of the initial crack was assumed perpendicular to the glass edge surface, starting in the vicinity of the maximum shear stress at the interface; the SIF computed for a crack length $a=0.2$ mm equals 0.58 MPa$\sqrt{\text {m}}$. The second crack path followed a line perpendicular to the direction of the maximum principal stresses, as the most unfavourable case for the effective glass resistance. In the observed refined area of $0.4\times 0.2$ mm, the angle equals $45^{\circ }$, measured counterclockwise from the glass edge surface. Further into the global model of the beam, the direction of the principal stresses gradually changes (Fig. 10b), resulting in the angle of (visible) crack propagation of $\sim 30^{\circ }$ (Fig. 8). The SIF computed along the $45^{\circ }$ inclined crack path reached a value of 0.73 MPa$\sqrt{\text {m}}$ for $a=0.28$ mm, which closely corresponds to the fracture toughness of glass ($K_{\text {Ic}}=0.75$ MPa$\sqrt{\text {m}}$), demonstrating that the applied 15 kN pre-load may initiate glass fracture. Although the assumed initial crack length is rather large for a polished glass edge [Lindqvist (2013) reported initial crack size in the range of 0.015 to 0.1 mm], a result in the same order of magnitude is considered acceptable, given a large scatter of glass edge quality which depends on the manufacturing process and varies among glass suppliers. Stress gradient along the crack path at $45^{\circ }$ is plotted in Fig. 12.

**Fig. 14:**
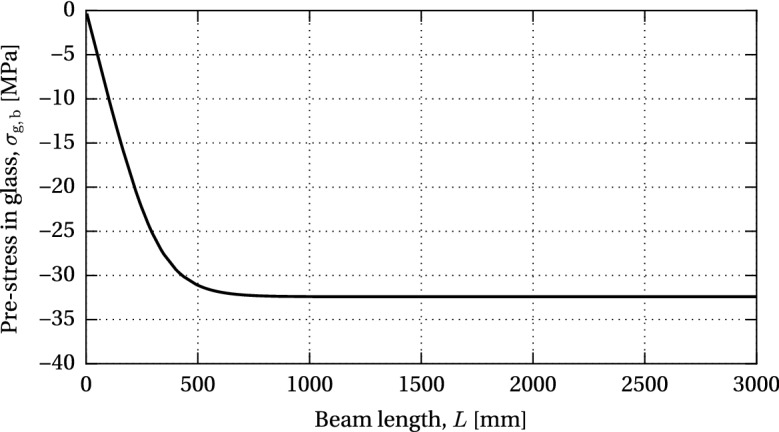
Relationship between the compressive pre-stress at the bottom glass edge and beam length at the verge of the adhesive failure

For the nominal beam properties and $\tau _{\text {g,max}}=8.28$ MPa, the analytical model yields a 7% lower maximum initial pre-load $P=14$ kN, at the verge of glass failure; the corresponding compressive pre-stress at the bottom glass edge, $\sigma _{\text {g,b}}$, equals -16.64 MPa, compared to -17.69 MPa obtained numerically. This can be explained by the conservative assumption of a fully rigid tendon-to-glass connection in the non-linear zone of the analytical model, while the FE model assumes linear-elastic adhesive behaviour over the entire bond length.

For a better qualification of the shear resistance of glass, *release tests* should be performed, in which the pre-load is gradually released into the beam through a bonded tendon, while monitoring the relative shear displacement along the interface. The results in terms of shear-slip curve upon initial glass failure (*softening*) could then be compared to the provided model, in order to validate the simplified linear softening law and the corresponding assumptions of the beam behaviour. This was, however, not performed in the scope of the present study.

## Application of the model in a parametric study

The analytical models AF and GF were applied in a parametric study in order to analyse the effectiveness of the investigated post-tensioned glass beam system, i.e. the achieved compressive pre-stress at the bottom glass edge, with varying geometric beam parameters—adhesive thickness, tendon height and beam length, and adhesive properties—strain limit (model AF) and shear modulus (model GF). The study was performed considering the nominal beam properties described in Sect. 3, varying one of the parameters. The initial pre-stress applied on the tendon at the verge of the adhesive/glass failure was calculated for each beam configuration.

**Fig. 15:**
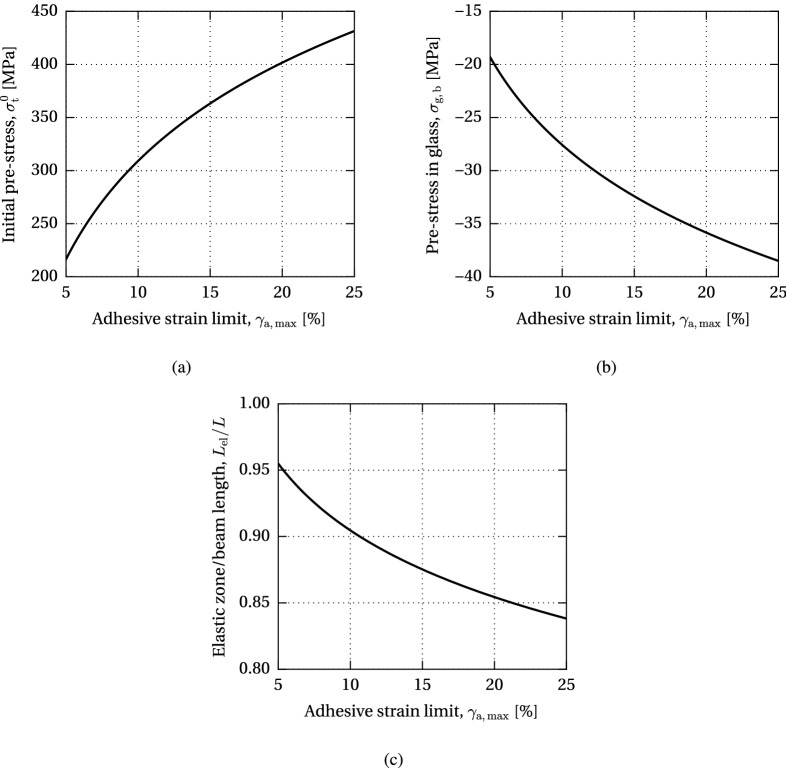
Variation of the allowable initial pre-stress level, resulting compressive pre-stress at the bottom glass edge and length of the elastic zone with respect to the adhesive strain limit (model AF)

**Fig. 16:**
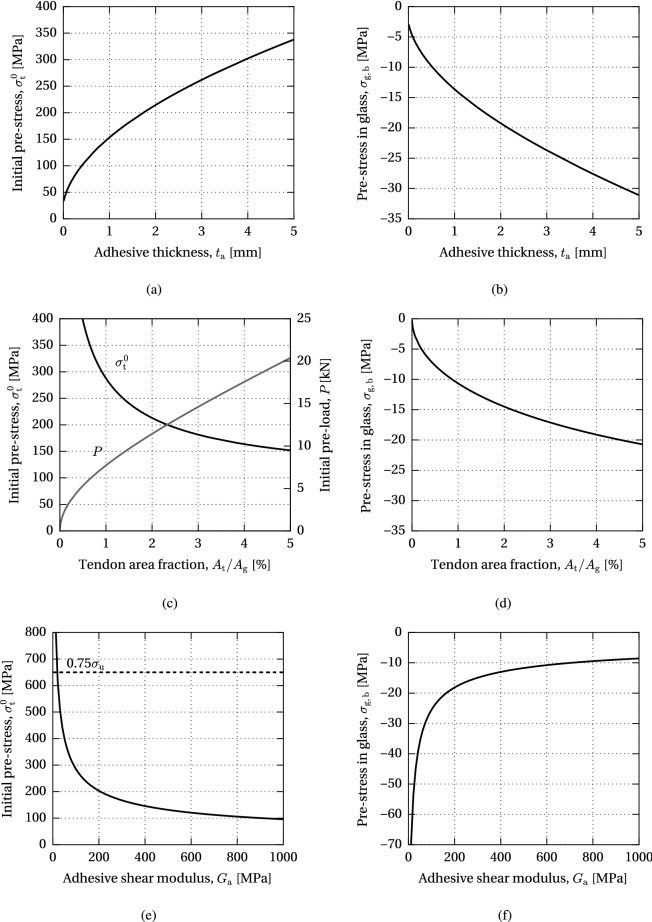
Variation of the allowable initial pre-stress level and resulting compressive pre-stress at the bottom glass edge with respect to the adhesive thickness, tendon area fraction and adhesive shear modulus (model GF)

### Model AF

The results of the parametric study of the maximum allowable pre-load governed by the adhesive failure are presented in Figs. 13, 14 and 15.

Figure 13 shows the initial pre-stress applied on the tendon, $\sigma ^{0}_{\text {t}}$, resulting compressive pre-stress in glass, $\sigma _{\text {g,b}}$, and ratio of the length of the elastic zone over the total length, $L_{\text {el}}/L$, with respect to the adhesive thickness and tendon area fraction, i.e. the cross-sectional area of the tendon, $A_{\text {t}}$, expressed as a percentage of the glass section, $A_{\text {g}}$. The limit of the initial pre-stress is set to 75% of the ultimate tensile strength of the tendon in order to avoid excessive stress relaxation. This amounts to 650 MPa for the stainless steel bars employed in this research (based on uniaxial tensile tests reported in Cupać (2017)). It can be seen that the allowable initial pre-stress level increases with the adhesive thickness, while it decreases with the increase in tendon area fraction (Fig. 13a, b). However, a larger tendon area yields a higher initial pre-load, *P* (Fig. 13b). Therefore, the compressive pre-stress at the bottom glass edge increases with both the adhesive thickness and tendon area fraction (Fig. 13c, d). An increase in both parameters results in a decrease in the ratio of the elastic length, i.e. an increase in the yield zone in the adhesive (Fig. 13e, f).

The variation of the compressive pre-stress in glass with respect to the beam length is shown in Fig. 14. An increase in the pre-stress can be seen up to *an effective bond length* at which the full pre-load is introduced into the glass; further increase in beam length does not affect the resulting compressive pre-stress. For the nominal dimensions of the investigated beam specimen, 99% of the maximum compressive pre-stress at mid-length is achieved with a beam length of $L=655$ mm.

Figure 15 shows the dependency of the post-tensioning system on the strain limit capacity of the applied adhesive. Similarly to the effect of the adhesive thickness, an increase in the adhesive strain limit enhances the maximum level of initial pre-stress in the tendon (Fig. 15a) and the achieved compressive pre-stress in glass (Fig. 15b), since the yielding of the adhesive increases the overall flexibility of the joint, diminishing excessive stress peaks at load introduction. Consequently, the ratio of the length of the elastic zone over the total beam length decreases with a higher yielding capacity of the adhesive (Fig. 15c).

### Model GF

The results of the parametric study of the maximum allowable pre-load governed by glass failure are shown in Fig. 16. It can be seen that the increasing adhesive thickness, $t_{\text {a}}$, positively influences the allowable initial pre-stress level, $\sigma ^{0}_{\text {t}}$, resulting in a higher compressive pre-stress at the bottom glass edge, $\sigma _{\text {g,b}}$, thus increasing the efficiency of the applied post-tensioning method (Fig. 16a, 16b). An increase in the tendon area relative to the cross-sectional area of glass, $A_{\text {t}}/A_{\text {g}}$, similarly provides a higher compressive pre-stress in glass, achieved through an increasing initial pre-load, *P*, which corresponds to a decrease in the initial pre-stress in the tendon (Fig. 16c, d). A significant increase in the efficiency of the system can be achieved by applying adhesive with a lower shear modulus, $G_{\text {a}}$, assuming that sufficiently high shear resistance of the adhesive is maintained (Fig. 16e, f).

## Discussion

Numerical verification of the proposed analytical model has shown that the model can be applied with sufficient accuracy for the prediction of short-term mechanical behaviour of post-tensioned glass beams, in terms of stress distribution in the tendon and the adhesive, and determination of the maximum compressive pre-stress that can be achieved in the glass without causing premature failure at pre-load introduction. The maximum compressive pre-stress predicted by the analytical model governed by adhesive strength (model AF) corresponds very closely to the results of the FEM (99%); in case of the model governed by the glass strength (model GF), the prediction is 6% lower than that obtained through numerical modelling. In the absence of an existing model for the shear failure of glass, an analogy with the softening of concrete in shear has been assumed. The shear strength of glass, $\tau _{\text {g,max}}$, has been determined based on a 2D numerical simulation of the release of pre-load. The obtained value has been verified by means of LEFM calculations and applied in the assumed simplified model for shear stress-slip relationship in glass. In order to improve the understanding of the mechanism of glass failure in shear and enhance the proposed analytical model, release tests should be performed by gradually releasing the pre-stress applied on the tendon until the first crack in the glass appears, while monitoring the relative slip between the glass and the tendon.

The parametric analysis of the effectiveness of the post-tensioned glass beam system has shown that the maximum level of compressive pre-stress in glass which can be attained through post-tensioning increases with tendon area fraction and increased flexibility of the bondline, achieved through increased adhesive thickness and strain capacity and lower adhesive stiffness.

The plastic deformation capacity of the adhesive has an important influence on the allowable pre-load level by reducing the shear stress peaks. Even with a very high plastic strain, the yield zone in the adhesive remains limited to a relatively small fraction of the total bond length (Fig. 15c). A complete absence of the plastic zone in the adhesive is explored in the model GF, governed by glass failure. The stress peaks are hence much higher; the assumed shear resistance of glass of 8.28 MPa is reached with a pre-load of 14 kN (analytically). For comparison, in the adhesive failure (AF) model, a shear resistance limit of the adhesive of 5.67 MPa is reached with a pre-load of 27.24 kN, considering a max. strain limit of 15%. Plastic deformation is in this sense very beneficial for the functioning of the system, while the limited yield zone does not pose a risk for the exploitation of the beam in bending, as long as the added shear deformation in bending is considered in the design. This, however, falls out of the scope of the present study which focuses on the pre-load introduction stage.

In order to determine the long-term behaviour of the proposed beam system, creep and relaxation behaviour of the constituent materials should be included in the model. In particular, given the viscoelastic nature of the adhesive, load-duration and temperature may affect the level of initially applied pre-load transferred into the glass, resulting in a lower efficiency of the system in the long term. Chemical compatibility of the adhesive and the interlayer material should also be investigated; given that the pre-load introduction and composite action fully rely on the adhesive bond, a lack of compatibility in the bonding zone may potentially jeopardise the entire system.

## Conclusions

The effectiveness of post-tensioning in enhancing the in-plane bending behaviour of a laminated glass beam with an adhesively bonded flat stainless steel tendon has been discussed taking into account the failure mechanisms that may cause premature failure of the system during post-tensioning or upon release of the applied pre-load from the post-tensioning set-up. Certain failure mechanisms, such as the rupture of the tendon and glass failure at the top glass edge, can be easily avoided with adequate detailing and simple structural verifications (the complexity may increase taking into account the effects of load duration and temperature).

Failure at load introduction has been investigated in more detail in order to determine a safe pre-load level that can be applied on the tendon prior to bonding, without initiating adhesive failure or glass fracture upon release. Allowable pre-load can be determined based on the provided analytical models, which showed good correlation with the numerical model of the release of pre-load, in terms of stress distribution in the tendon and the adhesive. Glass shear resistance has been verified by means of LEFM calculations; further investigations into shear-slip behaviour of glass by means of release tests are advised for better understanding of this failure mechanism.

Parametric study of the main beam parameters has shown that the effectiveness of the system, i.e. the level of the attained compressive pre-stress in glass, increases with adhesive thickness and tendon area fraction (for a uniform shear stress distribution across the adhesive thickness and negligible peeling stresses). In terms of the choice of the applied adhesive, high shear modulus and limited shear deformation capacity may lead to glass fracture at beam ends; therefore, increased flexibility of the joint should be sought through lower adhesive stiffness and plastic deformation of the adhesive in the load introduction region, as it will increase the efficiency of the system by distributing the stress peaks which may initiate premature failure.

## Footnotes

1 This method is referred to as *post-tensioning* (although the pre-load is only introduced into the glass upon curing of the adhesive bond) in analogy to post-tensioning in concrete, where *post* assumes an already cured concrete element. *Pre-tensioning* generally applies to the application of pre-stress on a tendon, followed by curing of the concrete element and finally release of pre-stress. The laminated glass beam is here considered an already formed structural element; the addition of the tendon therefore corresponds with *post-* rather than *pre*-processing.

2 Given a fairly high compressive strength of glass, a failure of glass in compression is unlikely to limit the allowable pre-load in practical applications, as long as constructive measures are taken during fabrication to avoid stability problems.

3 The overall beam width includes the width of the glass plies, $b_{\text {g,}i}$, and the thickness of the interlayers, $t_{\text {int}}$, and normally matches the width of the tendon, providing equal bonding surface on the glass beam and the tendon. For the calculation of beam resistance, only the thickness of the glass plies is taken into account, $b_{\text {g}} = \sum b_{\text {g,}i}$.

4 In crack mode I, the fracture energy (critical energy release rate) $G_{\text {Ic}}$ is related to the critical stress intensity factor $K_{\text {Ic}}$ by $G_{\text {Ic}} = K^{2}_{\text {Ic}} /E'$, where $E' = E$ for plane stress state, and $E' = E / (1-\nu ^{2})$ for plane strain state (Haldimann et al. 2008).

5 By Dassault Systémes Simulia Corp.
